# The Ethylates, or Caustic Alcohols

**Published:** 1894-07-28

**Authors:** Benjamin Ward Richardson


### THE ETHYLATES, OR CAUSTIC ALCOHOLS.

By Sir BENJAMIN WARD RICHARDSON, M.D., F.R.S.

IN my studies of the alcohol series of bodies, I was led to take up certain alcohols in which a new element is introduced in the place of one of the hydrogens. It has been a matter in dispute whether these new bodies should, strictly speaking, be called alcohols; but I do not myself see the objection, and as the term is expressive it had better be maintained. I studied especially sodium alcohol, or sodium ethylate; potassium alcohol, or potassium ethylate; sodium amylate, and potassium amylate. The composition of the first two, as compared with common ethylic alcohol, is as follows: Alcohol is written $C_2 H_5 HO$; sodium alcohol is written $C_2 H_5 NaO$; potassium alcohol is written $C_2 H_5 KO$, by which it will be seen how the elements of sodium and potassium enter into the composition of the new compounds.

The study included various specific investigations on the effect of substitution in remedies of the organic series. The question was what parts sodium and potassium would play when they were made to take the place of hydrogen in an ethyl compound.

I began by taking a specimen of sodium alcohol. The preparation is made by treating absolute alcohol with pure metallic sodium. A portion of absolute alcohol is placed in a glass beaker, and it is advisable at first to set the beaker in a larger basin containing cold water, to such an amount that the level of the water in the basin is equal to the level of the alcohol within the beaker. The next step consists in dropping small portions of bright metallic sodium into the alcohol. So soon as the sodium comes into contact with the alcohol there is brisk action with free escape of hydrogen, gently stirring with a glass rod and adding the sodium until all action ceases. When action is about to cease, if it be desired to make a concentrate solution the temperature of the water surrounding the beaker may with advantage be raised to 100 deg. Fahr. In the end there is obtained a thick whitish fluid consisting of a saturated solution of sodium alcohol, and if care be taken there can be often obtained the most beautiful set of crystals of this body, which will fill the space occupied in the beaker, and give no fluid whatever until solution is made by the addition of a little more alcohol. There is, however, no occasion to crystallise, so that I only name the fact of crystallisation that the maker of the ethylate may not be surprised if, when he is dealing with a concentrate solution, he sees the phenomenon suddenly developed.

So long as the sodium alcohol is kept apart from water it remains unchanged, but when water is added to it it immediately commences to decompose. The sodium is oxydised by the oxygen of the water forming sodium hydrate in fine caustic form, whilst the hydrogen goes back to the alcohol, reconstituting the common or ethylic alcohol. Observing this phenomenon I came to the conclusion that here was a very curious new remedy at my disposal. Suppose I should paint a moist surface of the skin with sodium alcohol, what ought naturally to occur? The water of the living tissue ought to decompose the solution; caustic soda ought to be produced which would immediately destroy the tissue, and alcohol reconstituted ought by its styptic effect to dry up and produce as a scaly structure that which had been broken up by the caustic.

I made some preliminary experiments with albuminous solutions, which convinced me that the theory thus formed was quite correct; and then I began to experiment on myself. I laid a layer, the size of half-a-crown, of caustic or sodium alcohol on my own arm, and let it remain. The effect was that it produced redness of the spot with tingling of a rather severe kind. In a few minutes the surface affected looked a little dark, felt a little hard, and had pouring out of it small drops or tears of water; finally, it produced a very firm scab, or scale, which in turn after two days began to loosen, and finally exfoliated, leaving what is called a new skin behind, but no scar or other mark. Thus the practice entirely confirmed the theory, and no better or demonstrable fact lies in the whole field of therapeutics.

The next step was to determine how this new caustic remedy could be applied in treatment. It was not a remedy that could be given internally, but it might, I thought, be very useful in cutaneous disease. The late Mr. John Gay had under his care a child suffering from a large vascular nævus. As many other means had been tried and had failed, he gave me permission to treat the nævus with sodium ethylate. I did so, producing on the nævus the precise effects I had observed on my own skin. The raised vascular surface was transformed into a large scab, the treatment giving very little pain, and, what was most satisfactory, causing no running or destruction of parts beyond those touched by the remedy, that is to say, none beyond the margin of the nævus itself. After a few days I detached the scale without difficulty, and finding still a red surface I painted it over once more with the ethylate, a more painful operation than that performed at first. The pain, however, soon subsided, and after two repetitions of the remedy there was complete recovery without leaving a trace of a scar. This was the original application of sodium ethylate in disease, and it claims, I venture to think, a most fitting illustration of the value of the substitution method in the matter of treatment.

Since the year 1870, when these researches with the caustic alcohols came to maturity, I have used sodium ethylate for the treatment of nævi in 103 cases, and in over 96 of these with complete and rapid success. The remedy, in fact, is so successful in the nævus of children I have never once known it fail where it was systematically as well as scientifically employed. When errors have been committed they have arisen from the circumstance that the solution has contained some water, or that the parts to which the solution has been applied have been washed with water, a proceeding always to be guarded against, with some others to which I will refer in my next communication.

